# Molecular Mapping of Oil Content and Fatty Acids Using Dense Genetic Maps in Groundnut (*Arachis hypogaea* L.)

**DOI:** 10.3389/fpls.2017.00794

**Published:** 2017-05-22

**Authors:** Yaduru Shasidhar, Manish K. Vishwakarma, Manish K. Pandey, Pasupuleti Janila, Murali T. Variath, Surendra S. Manohar, Shyam N. Nigam, Baozhu Guo, Rajeev K. Varshney

**Affiliations:** ^1^International Crops Research Institute for the Semi-Arid TropicsHyderabad, India; ^2^Department of Genetics, Osmania UniversityHyderabad, India; ^3^Crop Protection and Management Research Unit, Agricultural Research Service (USDA), TiftonGA, USA; ^4^School of Agriculture and Environment, University of Western Australia, CrawleyWA, Australia

**Keywords:** DArT/DArTseq, genetic map, QTLs, oil content, fatty acids, peanut

## Abstract

Enhancing seed oil content with desirable fatty acid composition is one of the most important objectives of groundnut breeding programs globally. Genomics-assisted breeding facilitates combining multiple traits faster, however, requires linked markers. In this context, we have developed two different F_2_ mapping populations, one for oil content (OC-population, ICGV 07368 × ICGV 06420) and another for fatty acid composition (FA-population, ICGV 06420 × SunOleic 95R). These two populations were phenotyped for respective traits and genotyped using Diversity Array Technology (DArT) and DArTseq genotyping platforms. Two genetic maps were developed with 854 (OC-population) and 1,435 (FA-population) marker loci with total map distance of 3,526 and 1,869 cM, respectively. Quantitative trait locus (QTL) analysis using genotyping and phenotyping data identified eight QTLs for oil content including two major QTLs, *qOc-A10* and *qOc-A02*, with 22.11 and 10.37% phenotypic variance explained (PVE), respectively. For seven different fatty acids, a total of 21 QTLs with 7.6–78.6% PVE were identified and 20 of these QTLs were of major effect. Two mutant alleles, *ahFAD2B* and *ahFAD2A*, also had 18.44 and 10.78% PVE for palmitic acid, in addition to oleic (33.8 and 17.4% PVE) and linoleic (41.0 and 19.5% PVE) acids. Furthermore, four QTL clusters harboring more than three QTLs for fatty acids were identified on the three LGs. The QTLs identified in this study could be further dissected for candidate gene discovery and development of diagnostic markers for breeding improved groundnut varieties with high oil content and desirable oil quality.

## Introduction

Groundnut or peanut (*Arachis hypogaea* L.) is one of the important oilseed crops in the world. It is grown in more than 100 countries with a global production of 42.4 Mt from an area of 25.7 Mha (FAO, 2014). More than 70% of the groundnut growing area comes under arid and semi-arid regions. Groundnut seeds contain edible oil (40–56%), protein (20–30%), carbohydrate (10–20%), and several nutritional components such as vitamin E, niacin, calcium, magnesium, phosphorus, zinc, iron, riboflavin, thiamine, and potassium (Dean et al., 2009). India and China are the largest groundnut producers and have a high consumption of groundnut, i.e., confectionery and as cooking oil. In western countries, groundnuts are majorly used in food industries for making peanut butter and confectionary and direct consumption as boiled, salted, and roasted nuts (Pandey et al., 2012; Varshney et al., 2013). It is estimated that 1% increase in the seed oil content increases the groundnut processer’s benefit by 7% (see Liao, 2003), indicating greater impact of oil content trait for farmers and traders.

Groundnut oil is a rich source of plant sterol especially β-sitosterol, which is known to have anticancer properties and reduce cholesterol levels upto 10–15% by inhibiting the cholesterol absorption (Awad and Fink, 2000). Groundnut oil is also a source of valuable antioxidants vitamin E, resveratrol, which neutralize the harmful free radicals, stresses and maintains cell membrane integrity (Meredith and Alfred, 2003). Along with the above beneficial features, groundnut oil is a high calorie diet, i.e., 100 g of oil provides 884 calories. The groundnut seed with high oil (≥50%) and high oleic acid (75–80%) contents can be an excellent alternative to canola and olive oil, which are currently costlier by 2.5 and 11 times, respectively. Currently selected countries such as Australia, Argentina, United States of America, Brazil, South Africa, and Israel are producing high oleic groundnuts on a large scale (Barkley et al., 2013) but such trend is likely to follow in other parts of the world.

The groundnut oil accounts 44–56% of the dry seed weight (Chen et al., 2010) and is composed of different fatty acids. Major fatty acids include oleic (C18:1), linoleic (C18:2), palmitic (C16:0), stearic (C18:0), arachidic (C20:0), behenic (C22:0), lignoceric (C24:0), and gadoleic acid (C20:1). Groundnut oil comprises about 80% unsaturated fatty acids (UFA) and 20% saturated fatty acids (SFA). UFA constitutes 42% mono UFA (MUFA), i.e., oleic acid while 37% poly UFA (PUFA), i.e., linoleic acid (Wang et al., 2015). Normal groundnut oil with high linoleic acid is vulnerable to oxidation, leading to unpleasant smell, taste and short shelf life of the oil and other groundnut products. The third major fatty acid palmitic acid (C16:0) is a SFA and contributes about 10% of the total oil content while the remaining 10% is contributed by minor fatty acids (Wang et al., 2015). In general, human consumption of the groundnut oil with high proportion of oleic acid and low proportion of linoleic acid (high oleic/linoleic acid ratio) is preferable because it cuts down the risk of cardiovascular disease (CVD) by reducing low-density lipoproteins (LDL) levels in the blood (Vassiliou et al., 2009). In this direction, identification of ‘F435,’ the first high oleic acid mutant, was the breakthrough for improving groundnut oil quality. The mutant ‘F435,’ with 80% oleic and 2% linoleic acid, has been deployed in breeding several groundnut cultivars with improved oil quality (Norden et al., 1987).

Fatty acid desaturase (FAD) enzyme facilitates the conversion of oleic acid to linoleic acid by adding a double bond to oleic acid. This enzyme is coded by two homologous genes (*ahFAD2A* and *ahFAD2B*) located on A and B subgenomes, respectively. Both *FAD* genes have 99% sequence homology and inactivation of this enzyme results in high oleic acid in mutants due to substitution (G:C to A:T) and insertion (A:T) of one base pair in FAD genes located on A and B subgenomes, respectively (Chen et al., 2010). These mutations lead to the accumulation of more oleic acid and decreasing linoleic and palmitic acid ensuring oil quality improvement by increasing shelf life. Keeping in mind the preferences of consumers and profitability to growers, groundnut breeding mainly focuses on developing improved varieties with desirable levels of oil content and other fatty acids, especially oleic, linoleic, and palmitic acids. Successful introgression of the *FAD* mutant alleles from SunOleic 95R with increased oleic acid has already been demonstrated using genomics-assisted breeding (GAB) approaches such as marker-assisted backcrossing (MABC) and marker-assisted selection (MAS) in both high and low oil containing genotypes (Chu et al., 2011; Janila et al., 2016b).

So far many studies have been conducted to decipher the biosynthesis pathway of fatty acids (Lung and Weselake, 2006) but very limited studies have been carried out in terms of genetics of fatty acids synthesis and its regulation (Wang et al., 2015). Previous genetic studies on groundnut oil trait suggested that it was highly influenced by genotype, environment, and genotype × environment interaction and was also positively correlated with soil pH, iron content, and oleic/linoleic acid (O/L) ratio (Dwivedi et al., 1993). Although the fatty acid composition varies with groundnut growth habits, the oil content is independent of them (Bansal et al., 1993). Oil content synthesis and accumulation is guided by complex pathways and it is considered a quantitative trait (Dwivedi et al., 1993).

In order to perform high resolution trait mapping, it is essential to generate dense genetic maps and good phenotyping data (Pandey et al., 2016). A high density linkage map plays a very crucial role in the identification of the QTLs for any crop, especially for complex polygenic traits (Guo et al., 2013, 2016; Varshney et al., 2013). To date, various QTL analysis studies for oil content and fatty acids have been conducted using early generation markers like SSR (Selvaraj et al., 2009; Sarvamangala et al., 2011; Pandey et al., 2014; Wang et al., 2015), but the marker density of these genetic maps were still low. With recent advances in availability of reference genomes for diploid progenitors of groundnut (Bertioli et al., 2016; Chen et al., 2016), the genotyping platforms and availability of sequencing technology along with array-based genotyping methods (Pandey et al., 2017), it has become easy to generate thousands of data points for conducting high resolution trait mapping (Pandey et al., 2016). Diversity Arrays Technology (DArT) and sequence based (DArTseq) platform are the advanced genotyping technologies, which were successfully used in diverse crops to study various traits (Raman et al., 2013). The power of this technique lies in the reduction of genome complexity by restriction enzymes followed by microarray hybridization to assay markers across the genome. This methodology clearly increases the probability to generate polymorphic data points in the present study. GAB has already demonstrated its strength in accelerated improvement of target traits in groundnut and therefore, availability of linked markers for oil content and other minor fatty acids will help in breeding the improved varieties with a desirable combination of oil content and fatty acid composition. The present study reports the development of two dense genetic maps and their use in identifying the QTLs for oil content and fatty acids in groundnut.

## Materials and Methods

### Plant Materials

Two F_2_ populations, i.e., one for oil content (ICGV 07368 × ICGV 06420) and the other for fatty acids (ICGV 06420 × SunOleic 95R), were developed at ICRISAT, Patancheru, India. These two populations have been referred to as OC-population and FA-population, respectively, throughout the article. The parent “ICGV 07368” is a high yielding variety with low oil content (45%) and the parent “ICGV 06420” is a drought tolerant variety with high oil content (55%) and normal fatty acid profile. The parent “SunOleic 95R” is the first high oleic groundnut variety released in the USA and carries *FAD* mutant alleles in A and B subgenomes (Gorbet and Knauft, 1997). The hybridity of F_1_ plants of both the populations was confirmed using SSR markers and were selfed to develop F_2_ populations.

### DNA Extraction and Genotyping with SSR, DArT, and DArTseq Markers

DNA from both mapping populations was extracted from 10 to 15 days young F_2_ plants along with parents using the modified cetyltrimethylammonium bromide (CTAB) extraction method (Mace et al., 2003). The DNA quality and quantity were checked on 0.8% agarose and DNA was diluted to 5 ng/μl of working concentrations for genotyping work.

The genotyping with SSR markers was performed following the methods explained in Varshney et al. (2009). PCR products were resolved on 1.5% agarose gel for confirming the amplification. The forward primers were dye labeled with FAM, VIC, and NED, which were detected as blue, green, and black color peaks, respectively (Applied Biosystems, USA). The PCR products were denatured and capillary separated with ABI 3700 automatic DNA sequencer (Applied Biosystems, USA) and GeneMapper Software v (Applied Biosystems, USA) was used to analyze the results.

Both F_2_ populations along with their parents were also genotyped with a DArT array consisting of 15,360 features at Diversity Arrays Technology Pty Ltd. (DArT P/L), Australia. The detailed method of genotyping is available at the website^1^ and is also described in Pandey et al. (2014). DArTseq procedure is quite similar to DArT genotyping and involves sequencing based method along with some steps of DArT as described in Vishwakarma et al. (2016). All polymorphic sequences of the DArT and DArTseq markers generated from the parental lines of the F_2_ populations were scored as presence vs. absence and used to construct genetic maps.

### Phenotyping of Oil Content and Fatty Acids

Phenotyping data was generated on 184 individuals of OC-population for oil content while on 179 individuals of FA-population for different fatty acid compositions, namely oleic (C18:1), linoleic (C18:2), palmitic (C16:0), stearic (C18:0), arachidic (C20:0), behenic (C22:0), and lignoceric (C24:0) at ICRISAT, Patancheru, India. The oil content and fatty acid composition were estimated using near infrared reflectance spectroscopy (NIRS) (Model XDS RCA, FOSS Analytical AB, Sweden, Denmark) (Sundaram et al., 2010). Oil content estimation was also done using the Soxhlet method following the protocol of Sharma et al. (2002). In addition to NIRS, the fatty acids were also estimated by gas chromatography (GC) (Shimadzu GG-9A GLC unit, Tokyo, Japan), following the protocol of Metcalf et al. (1966).

### Phenotypic Data Analysis

Phenotyping data was analyzed using SPSS software^2^. Association among different fatty acids was established by Pearson correlation and calculating a two-tailed *P*-value with 95% confidence intervals. The Chi-square test was performed to check the goodness of fit of the segregation ratios of three major fatty acids, i.e., oleic, linoleic, and palmitic acids.

### Construction of Genetic Maps

The genetic maps were constructed using JoinMap version 4 (Van Ooijen, 2006). The Kosambi map function was used for genetic map construction and calculation of map distance from recombination fractions. The markers were integrated into the framework genetic maps by applying the independence LOD (logarithm of the odds), with LOD threshold ranging from 2.0 to 20.0 with a minimum recombination frequency (∂) threshold of 40%. In the case of OC-population, initially the framework genetic map was constructed with polymorphic SSR markers and then DArT and DArTseq marker data were integrated for developing dense genetic maps. For FA-population, the genetic map was constructed using DArT and DArTseq polymorphic loci and then *ahFAD2A* and *ahFAD2B* mutant allele specific loci were also integrated. MapChart was used to draw a final genetic map for better visualization (Voorrips, 2002).

### Quantitative Trait Locus (QTL) Analysis

The genetic map information together with phenotyping data was used for the identification of QTLs for oil content and fatty acids using ICIM mapping software version 4.0 (Wang et al., 2012). Inclusive Composite Interval Mapping (ICIM) for QTLs with additive (one-dimensional scanning, ICIM-ADD) method with 1cM step and 0.001 probability mapping parameters in stepwise regression were employed in QTL analysis. The LOD threshold score of 3.0 as minimum significance level with 1000 permutations was set manually.

## Results

### Phenotypic Variation for Oil Content and Fatty Acids

**FIGURE 1:**
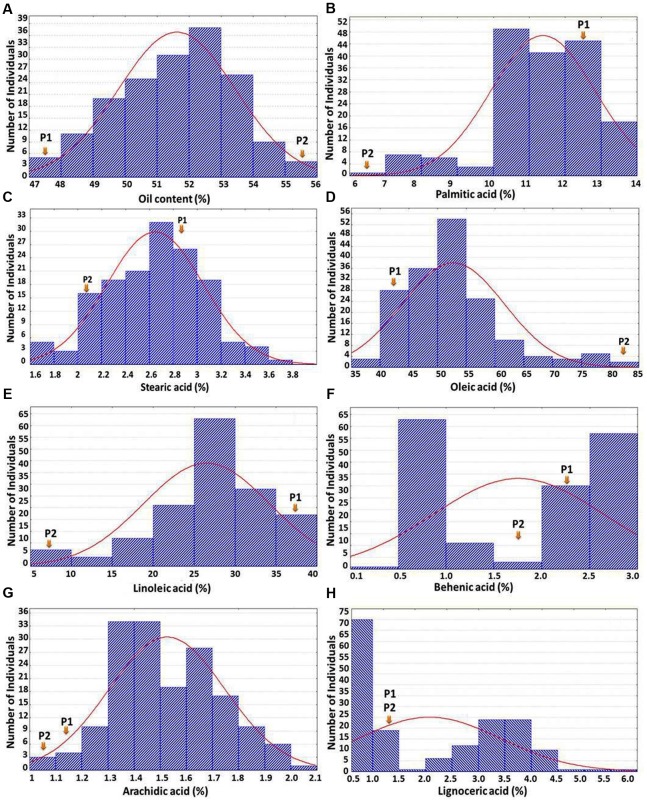
**Frequency distribution of oil content and seven different fatty acids.** The *X*-axis shows the percentage of the trait and the *Y*-axis represents the number of individuals in F_2_ population. P1 and P2 represent the parents ICGV 07368 and ICGV 06420, respectively, in **(A)**; while P1 and P2 represent the parents ICGV 06420 and SunOleic 95R, respectively, in **(B–H)**.

The OC-population derived from cross ICGV 07368 (45%) × ICGV 06420 (55%) showed good segregation for oil content ranging from 47.2 to 55.7% with an average oil content of 51.6% (**Figure 1A**). In FA-population, the parental genotype ‘SunOleic 95R’ possessed desirable major fatty acid composition (82% oleic acid, 3% linoleic acid, and 6% palmitic acid), while ‘ICGV 06420’ possessed undesirable major fatty acid composition (45% oleic acid, 31% linoleic acid, and 12% palmitic acid) along with minor fatty acids. The phenotypic variability of fatty acids in the F_2_ population ranged from 35.8 to 81.8% for oleic acid, 4.6 to 39.1% for linoleic acid, 0.2 to 3% for behenic acid, 0.2 to 4.5% for lignoceric acid, 6.6 to 13.9% for palmitic acid, 1.7 to 4.3% for stearic acid, and 1.0 to 2.6% for arachidic acid (**Figures 1B–H**). Phenotyping data on OC-population showed a normal distribution for oil content. In the case of oleic acid, linoleic acid, palmitic and stearic acids, although normal distribution is followed, but the curve is shifted to one side with few transgressive segregants (**Figures 1B–E**). In the case of behenic acid and lignoceric acids, although the phenotypic variability between the parental genotypes of the mapping population was very less but we have observed a significant number of transgressive segregants toward either side of the phenotypic extreme. Similarly, higher frequency of individuals with transgressive segregation was observed for arachidic acid in the FA-population (**Figure 1G**).

### Correlation between Different Fatty Acids and Genetic Nature of Major Fatty Acids

**FIGURE 2:**
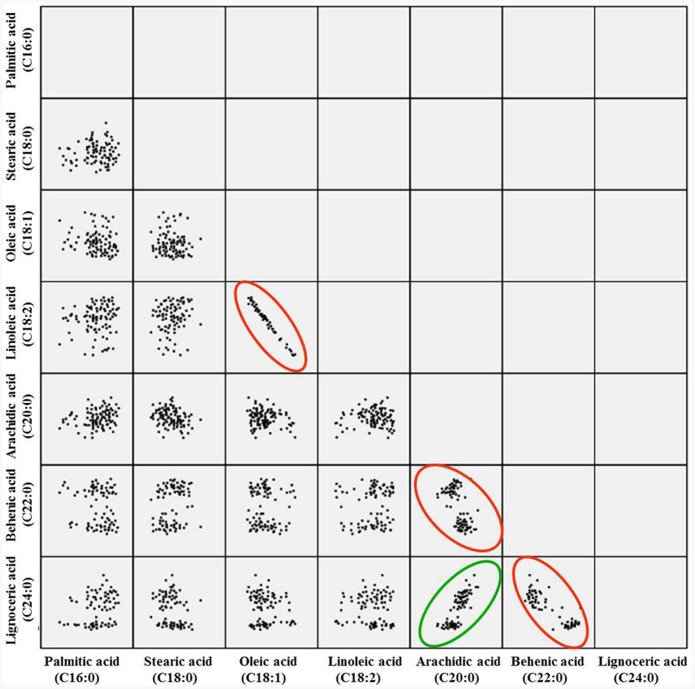
**Pairwise correlation of the seven different fatty acids in F_2_ population.** The red circle indicates negative correlation, while green indicates the positive correlation.

The correlation analysis clearly indicated a significant and negative correlation of oleic acid [C18:1] with linoleic acid [C18:2] (*r* = -0.701, *P* < 0.0001), stearic acid [C18:0] (*r* = -0.218, *P* < 0.001), and arachidic acid [C20:0] (*r* = -0.331, *P* < 0.0001) (**Table 1** and **Figure 2**). A significant positive correlation was observed for linoleic with palmitic acid [C16:0] (*r* = 0.888, *P* < 0.0001) and stearic acid [C18:0] (*r* = 0.239, *P* < 0.001). Palmitic acid was negatively correlated with oleic acid (*r* = -0.475, *P* < 0.001) and behenic acid [C22:0] (*r* = -0.155, *P* < 0.01) (**Table 1** and **Figure 2**). Chi square (χ^2^) analysis for oleic acid on F_2_ progeny revealed digenic 15:1 (low:high) segregations. Similarly, linoleic acid and palmitic acid also followed digenic 15:1 (high:low) ratio (**Table 2**).

**Table 1:** Pairwise correlation among the six different fatty acids in FA-population (ICGV 06420 × SunOleic 95R).

	Palmitic acid (C16:0)	Stearic acid (C18:0)	Oleic acid (C18:1)	Linoleic acid (C18:2)	Arachidic acid (C20:0)	Behenic acid (C22:0)	Lignoceric acid (C24:0)
Palmitic acid (C16:0)	1						
Stearic acid (C18:0)	0.052	1					
Oleic acid (C18:1)	-0.475***	-0.218**	1				
Linoleic acid (C18:2)	0.888***	0.239**	-0.701***	1			
Arachidic acid (C20:0)	-0.048	0.016	-0.331***	-0.121	1		
Behenic acid (C22:0)	-0.155*	0.514***	0.056	0.100	-0.627^∗∗∗^	1	
Lignoceric acid (C24:0)	0.282***	-0.460***	-0.004	-0.002	0.649^∗∗∗^	-0.891^∗∗∗^	1


*^∗^, ^∗∗^, ^∗∗∗^ = statistically significant at 0.01, 0.001, and 0.0001 probability level, respectively.*

**Table 2:** Test of segregation for three major fatty acids.

FA-population	Fatty acid range (%)	Observed normal	Observed high	Observed low	Expected normal (15:1)	Expected low	Expected high (15:1)	χ^2^ (15:1)	Expected normal (3:1)	Expected high (3:1)	χ^2^ (3:1)
Oleic acid	35.8–81.7	160	10	–	159.35	NA	10.65	0.010	128.25	42.75	31.44^∗∗∗^
Linoleic acid	2.5–38	160	–	10	159.35	10.65	–	0.010	128.25	42.75	31.44^∗∗∗^
Palmitic acid	6.6–13.9	160	–	10	159.35	10.65	–	0.010	128.25	42.75	31.44^∗∗∗^


*^∗∗∗^ = difference is statistically significant at 0.001 probability level.*

### Effect of Mutant FAD Alleles on Different Fatty Acids

In this study, the parent genotype ‘ICGV 06420’ had heterozygous mutant *FAD* allele (ol_1_Ol_1_) in the A subgenome and homozygous dominant mutant *FAD* allele in the B subgenome (Ol_2_Ol_2_), and the other parent ‘SunOleic 95R’ had homozygous recessive mutant *FAD* alleles at both the loci of two subgenomes (ol_1_ol_1_ol_2_ol_2_). Genotyping of the FA-population for *ahFAD* mutant alleles yielded six allelic combinations (ol_1_ol_1_ Ol_2_Ol_2,_ ol_1_ol_1_ ol_2_ol_2,_ ol_1_ol_1_ Ol_2_ol_2,_ ol_1_Ol_1_ Ol_2_Ol_2,_ ol_1_Ol_1_ ol_2_ol_2_, and ol_1_Ol_1_ Ol_2_ol_2_) (**Table 3**). As expected the homozygous mutant combination (ol_1_ol_1_ ol_2_ol_2_) showed the highest average of oleic acid (65%) and the lowest for linoleic (15.6%) and palmitic (9.7%) acids. It was also observed that the heterozygous mutant *ahFAD2B* (Ol_2_ol_2_) allele also contributed to the significant increase in oleic acid (60%) and reduced linoleic (20.8%), and palmitic (9.7%) acids from the normal fatty acid profile.

**Table 3:** The fatty acid mean composition (%) for the genotypes detected in the F_2_ segregating population (ICGV 06420 × SunOleic 95R).

Mutant FAD2A allele in A subgenome	Mutant FAD2B allele in B subgenome	Palmitic acid (C16:0)	Stearic acid (C18:0)	Oleic acid (C18:1)	Linoleic acid (C18:2)	Arachidic acid (C20:0)	Behenic acid (C22:0)	Lignoceric acid (C24:0)
ol_1_ol_1_	Ol_2_Ol_2_	11.07	2.85	51.87	27.73	1.42	2.43	1.13
ol_1_ol_1_	ol_2_ol_2_	9.68	2.59	65.02	15.63	1.45	1.83	2.13
ol_1_ol_1_	Ol_2_ol_2_	9.69	2.22	60.14	20.87	1.34	2.00	1.40
Ol_1_ol_1_	Ol_2_Ol_2_	12.79	2.68	49.05	29.37	1.58	1.49	2.47
Ol_1_ol_1_	ol_2_ol_2_	10.56	2.57	54.13	24.92	1.58	1.50	2.57
Ol_1_ol_1_	Ol_2_ol_2_	11.34	2.49	51.44	27.13	1.64	1.25	2.86


**FIGURE 3:**
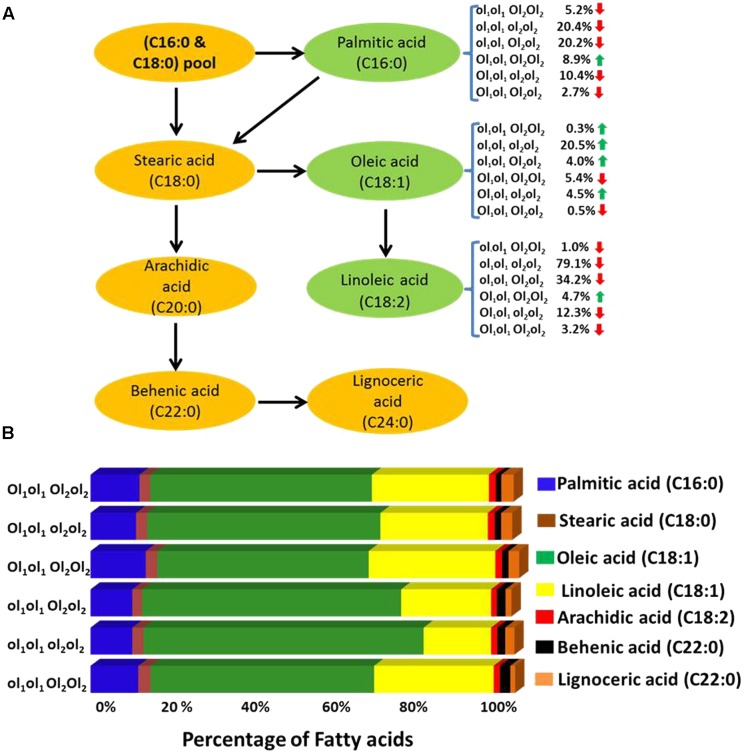
**(A)** The effect of *FAD* mutant allele combinations of different fatty acids. The ol_1_ and Ol_1_ represent the mutant and the wild type allele, respectively, of A subgenome while ol_2_ and Ol_2_ represent the mutant and the wild type allele, respectively, of B subgenome. Red and green arrow indicates the decrease and increase of the fatty acid percentage levels, respectively. **(B)** Mean fatty acid composition (%) in plants possessing different mutant FAD allele (mutant and wild) combinations in fatty acid (FA)-population.

Few genotypes with the homozygous combination of both mutant *FAD* alleles (*ahFAD2A* and *ahFAD2B*) showed a high oleic acid content of 81.7%, which is similar to ‘SunOleic 95R,’ the high oleic parent. The dosage of the *FAD* mutant alleles clearly defined the composition of three major fatty acids, namely oleic, linoleic, and palmitic acids (**Figure 3** and **Table 3**). Genotypes with homozygous *ahFAD* allele mutation in A and B subgenomes (ol_1_ol_1_ ol_2_ol_2_) showed an average increase in the oleic acid by 20.5%, decrease in the linoleic acid by 79% and in palmitic acid by 20% (**Figure 3**). Although heterozygous (Ol_1_ol_1_ Ol_2_ol_2_) mutant FAD allele combination was found influencing the major fatty acids, but in lesser amounts because of decrease in dosage of number of mutant alleles. Further, the variation in the other minor fatty acids was less significantly recorded. None of the *FAD* allele combinations influenced the stearic acid levels significantly.

### Genetic Maps for Two Populations

A polymorphic survey of parental lines of OC-population with 250 SSR markers yielded 55 polymorphic markers. Genotyping data generated for these polymorphic SSR markers were used for development of framework genetic map for OC-population. In addition to SSRs, further genotyping of OC-population with 15,360 DArT and DArTseq yielded genotyping data for 2,238 polymorphic loci. A total of 1,384 polymorphic loci were either highly distorted or were having missing data, therefore, not included in constructing the genetic map. Genetic map construction with these polymorphic markers resulted in the development of a genetic map with 854 mapped loci (799 DArT and DArTseq markers and 55 SSRs) into 20 linkage groups (LGs) (**Table 4**, **Supplementary Figure S1**, and **Table S1**). This genetic map covered a total genome distance of 3,525.8 cM and achieved a density of 4.1 cM/loci ranging from 20 (A10) to 79 (B04) loci per LG. Similarly, the length of the LGs ranged from 94.6 cM (B06) to 288.3 cM (B09). In case of FA-population, genotyping data with 15,360 DArT/DArTseq features yielded 1,725 (655 DArT and 1,070 DArTseq) polymorphic loci. Of these polymorphic loci, 1,435 DArT/DArTseq marker loci, including two *ahFAD2A* and *ahFAD2B* alleles, were mapped on 20 LGs spanning a total map distance of 1,869.17 cM with an average inter marker distance of 1.3 cM. The number of mapped loci ranged from 16 (B07) to 281 (A05) loci and length of the LGs ranged from 26.0 cM (B07) to 153.8 cM (B04) (**Table 4**, **Supplementary Figure S2**, and **Table S2**).

**Table 4:** Details of the linkage maps for populations ICGV 07368 × ICGV 06420 (oil content) and ICGV 06420 × SunOleic 95R (fatty acids).

Linkage group (LG)	Oil content population (ICGV 07368 × ICGV 06420)			Fatty acid population (ICGV 06420 × SunOleic 95R)		
		
	Mapped loci	Map distance (cM)	Average inter marker distance (cM/loci)	Mapped loci	Map distance (cM)	Average inter marker distance (cM/loci)
A01	50	145.4	2.9	52	40.7	0.7
A02	50	176.1	3.5	43	68.4	1.5
A03	21	105.4	3.9	57	88.5	1.5
A04	42	208.1	4.4	78	51.4	0.6
A05	61	252.5	4.1	281	107.6	0.3
A06	43	202.7	4.7	44	123.5	2.8
A07	28	149.2	5.3	123	125.4	1.0
A08	70	279.9	4.0	72	105	1.4
A09	32	124.0	5.0	73	146.6	2.0
A10	20	118.4	5.9	30	77.3	2.5
B01	38	157.9	4.2	35	83.5	2.3
B02	28	144.5	5.2	149	110.1	0.7
B03	58	259.3	4.5	42	63.2	1.5
B04	79	247.0	3.1	54	153.8	2.8
B05	26	112.4	4.3	66	68.9	1.0
B06	26	94.6	3.6	102	82.6	0.8
B07	45	114.7	2.5	16	26	1.6
B08	26	168.8	6.5	28	133.1	4.7
B09	65	288.3	4.9	60	135	2.2
B10	46	176.6	3.8	30	77.9	2.5
**Total**	**854**	**3,526**	**4.1**	**1,435**	**1,869**	**1.3**


### QTLs for Oil Content and Fatty Acids

**FIGURE 4:**
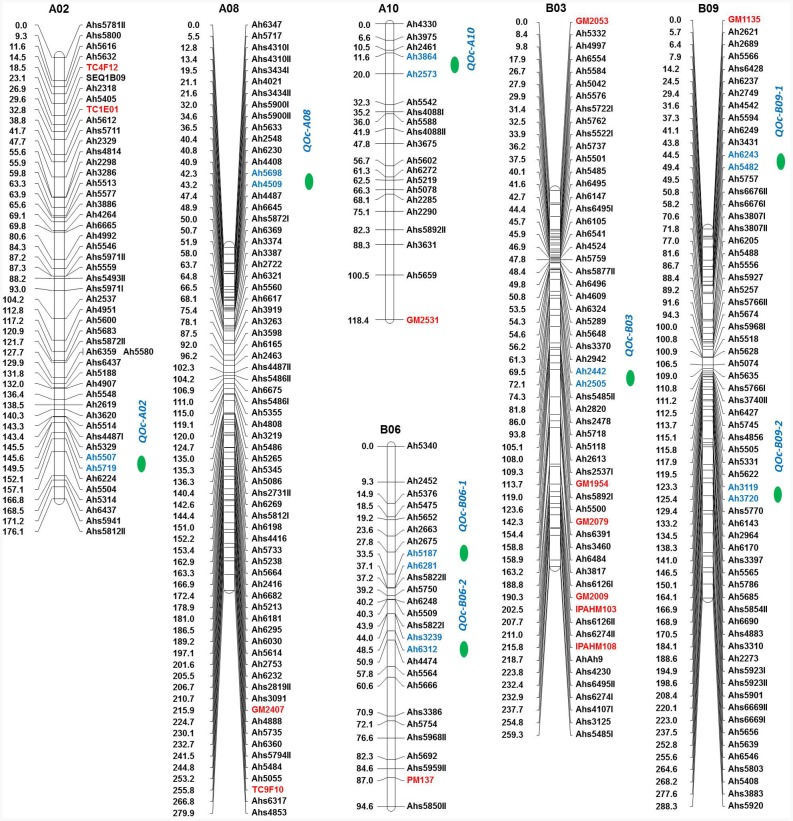
**Genetic map of the oil content (OC) population showing quantitative trait locus (QTLs) for oil content.** For each linkage group (LG), marker loci are given on the right side of the bar and marker positions (in cM) are mentioned on the left side. Markers with red color indicate the SSRs while markers in blue color represent the flanking markers of the identified QTL region. QTL location is indicated with a green color circle.

The QTL analysis identified eight QTLs for oil content (**Table 5** and **Figure 4**) with PVE ranging from 5.67 to 22.11%. Of these eight QTLs, two are having major effect. The first major QTL ‘*qOc-A10*’ explained 22.11% PVE with a LOD of 13.21 located on A10 and the second major QTL ‘*qOc-A02*’ explained 10.37% PVE with a LOD 4.8 mapped on A02. Of the six minor-effect QTLs, two QTLs, *qOc-B09-1* and *qOc-B09-2*, were mapped on B09 explaining PVE 8.40 and 7.83%, respectively. Two QTLs, *qOc-B06-1* and *qOc-B06-2*, were detected on B06 explaining 6.19 and 7.13% PVE, respectively. The remaining two QTLs, *qOc-A08* and *qOc-B03*, were mapped on A08 and B03 with 7.05 and 5.67% PVE, respectively.

**Table 5:** Features of QTLs identified for oil content and fatty acids.

S.No.		QTL	Linkage group	Flanking markers	QTLs region (cM)	LOD value	Phenotypic variance explained (%)

	Oil content						
1	Oil content	*qOc-A02*	A02	Ah5507 – Ah5719	145–149	4.8	10.4
2		*qOc-B09-1*	B09	Ah6243 – Ah5482	44–49	3.8	8.4
3		*qOc-B09-2*	B09	Ah3119 – Ah3720	123–125	3.0	7.8
4		*qOc-A08*	A08	Ah5698 – Ah4509	42–43	3.4	7.0
5		*qOc-A10*	A10	Ah3864 – Ah2573	11–20	13.2	22.1
6		*qOc-B03*	B03	Ah2442 – Ah2505	69–72	4.4	5.6
7		*qOc-B06-1*	B06	Ah5187 – Ah6281	33–37	4.6	6.2
8		*qOc-B06-2*	B06	Ahs3239 – Ah6312	43–48	4.4	7.1

	**Fatty acids**						

9	Oleic acid	*qOle-*A09*-1*	A09	Ah3819 – FAD2A	13–24	20.3	17.4
10		*qOle-*A09*-2*	A09	Ah5452 – Ah6158	76–77	25.2	34.2
11		*qOle-*B09	B09	FAD2B – Ah3931	82–87	36.1	33.8
12	Linoleic acid	*qLin-*A09*-1*	A09	Ah3819 – FAD2A	13–24	6.2	19.5
13		*qLin-*A09*-2*	A09	Ah5853 – Ahs6334II	99–107	4.0	12.1
14		*qLin* B09	B09	FAD2B – Ah3931	82–87	22.4	41.0
15	Palmitic acid	*qPal-*A09	A09	Ah3819 – FAD2A	13–24	3.7	10.7
16		*qPal-*B09	B09	FAD2B – Ah3931	82–87	16.6	18.4
17		*qPal-A08*	A08	Ah4653 – Ah4264	23–25	17.9	20.1
18		*qPal-A10*	A10	Ah4042 – Ah4430	61–71	5.2	10.0
19		*qPal-B04*	B04	Ahs2963 – Ahs3184I	105–153	3.9	10.0
20	Lignoceric acid	*qLig-*A09	A09	Ah5241 – Ah3376	95–97	10.4	12.1
21		*qLig-A05-1*	A05	Ah4275 – Ah6374	22–23	14.0	13.4
22		*qLig-A05-2*	A05	Ahs3122II – Ahs4487II	76–77	12.3	11.8
23		*qLig-A08*	A08	Ah5275 – Ah3064	70–75	3.07	10.5
24		*qLig-B01*	B01	Ah4564 – Ah6355	30–31	3.07	10.3
25	Behenic acid	*qBeh-A05*	A05	Ahs4487II – Ahs3271I	77–78	17.8	12.0
26		*qBeh-*A09	A09	Ah5497 – Ah2642	92–94	8.6	8.4
27	Arachidic acid	*qAra-A05*	A05	Ahs3122II – Ahs4487II	76–77	17.7	23.4
28		*qAra-A07*	A07	Ah4597 – Ah6254	84–85	4.8	12.4
29	Stearic acid	*qSte-*A09	A09	Ah5452 – Ah6158	76–77	191	78.6


**FIGURE 5:**
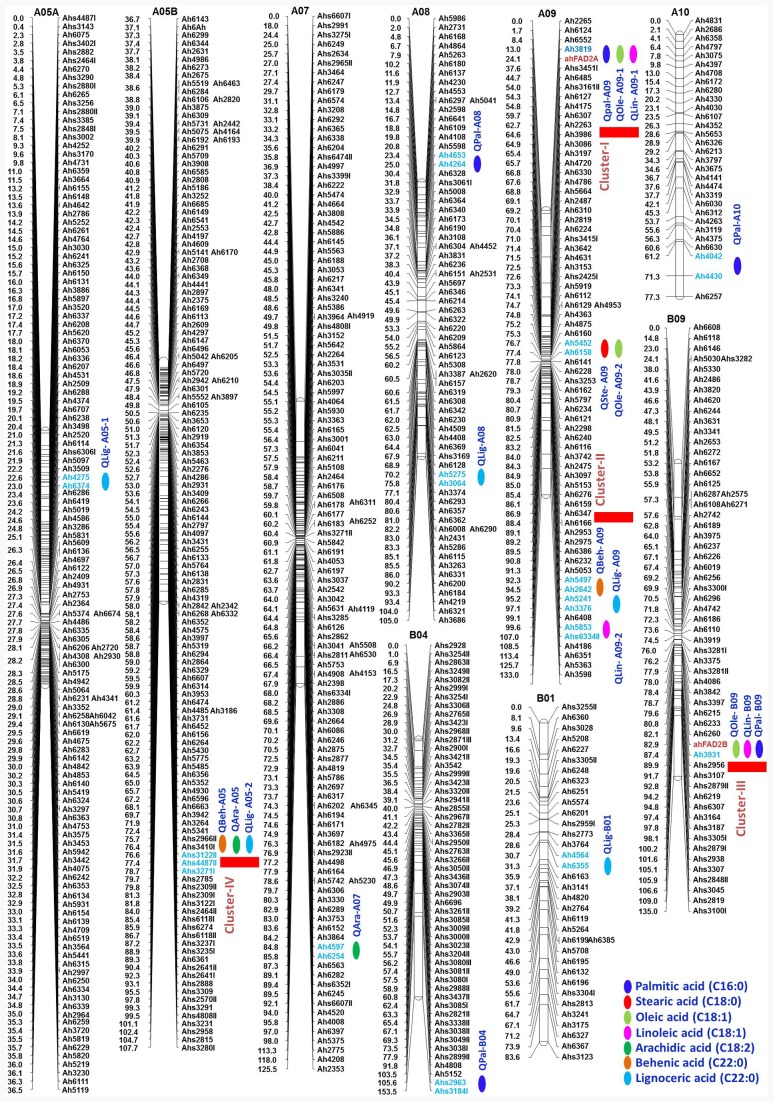
**Genetic map of the FA-population showing QTLs for different fatty acids.** For each LG, marker loci are given on the right side of the bar and marker positions (in cM) are mentioned on the left side. Markers with red color indicate the SSRs while markers in blue color represent the flanking markers of the identified QTL region. QTL location is indicated with a green color circle.

The QTL analysis identified 21 QTLs for seven fatty acids in FA-population, of which 20 were the major effect (**Table 5** and **Figure 5**). For oleic acid, three major QTLs namely, *qOle-*A09*-1* (A09), *qOle-A09-2* (A09), and *qOle-B09* (B09), were identified explaining 17.4, 34.2, and 33.8% PVE, respectively. For linoleic acid, two major QTLs were identified on A09 viz. *qLin-A09-1* and *qLin-A09-2* explaining 19.5 and 12.1% PVE. The third major QTL *qLin-*B09 mapped on B09 could explain 41% PVE. For palmitic acid, five QTLs, with a combined 69.2% PVE, were identified on five different LGs (A08, A09, A10, B04, and B09). For arachidic acid, two QTLs were detected, i.e., one each on A05 and A07 explaining 23.4 and 12.4% PVE, respectively. For behenic acid, one minor and one major effect QTL were detected on A09 and A05 with PVE 8.4 and 12.0%, respectively. In the case of lignoceric acid, five QTLs were identified on four LGs (A05, A07, A09, and B01) explaining altogether 58% PVE. Interestingly, for steric acid, one major QTL namely *qSte-A09* with 78.6% PVE was mapped on A09.

Of the 21 QTLs identified in FA-population, A09 holds the highest number of eight QTLs followed by four QTLs on A05, three QTLs on B09, two QTLs on A08, and one QTL each on A07, A10, B01, and B04. The *ahFAD2A* and *ahFAD2B* alleles were mapped onto A09 and B09, respectively, and showed high influence on many fatty acids. Four genomic regions were also identified, where a minimum of three QTLs for fatty acids were mapped. Two QTL clusters were identified on A09, i.e., Cluster-I between Ah3819-ahFAD2A and Cluster-II between Ah5497-Ah6334I. Cluster-I harbored QTLs for palmitic, oleic, and linoleic acids, while Cluster-II for lignoceric, linoleic, and behenic acids. Cluster-III identified on B09 between ahFAD2B-Ah3931 harboring QTLs for palmitic, oleic, and linoleic acids, while Cluster-IV on A05 between Ahs3122II-Ahs4487II harbored QTLs for arachidic, lignoceric, and behenic acids.

## Discussion

Genomics-assisted breeding has facilitated accelerated development of oil quality (Chu et al., 2011; Janila et al., 2016a) and disease resistance (Varshney et al., 2014) in groundnut. This study successfully developed dense genetic maps and identified genomic regions and linked markers for oil content and fatty acids. These genomic regions in the present study will facilitate further gene discovery and marker development for these traits in groundnut.

### Genetic Behavior of Oil Content and Fatty Acids

Polygenic traits are quantitative in nature and do not follow distinct or absolute values; instead, there will be a gradient of expression with little variation. The present study showed continuous variation for oil content, indicating its polygenic nature similar to the other major oilseed crops such as soybean, rapeseed, mustard, etc. (Singh et al., 2013; Wang et al., 2013; Akond et al., 2014). The polygenic nature of oil content was also earlier reported in different recombinant inbred line (RIL) populations in groundnut (Sarvamangala et al., 2011; Pandey et al., 2014).

Mutant *FAD* alleles are known for controlling oleic and linoleic acids, *FAD2B* being a higher phenotypic contributor as compared to *FAD2A* (Pandey et al., 2014). The present study showed that major fatty acids, namely oleic, linoleic, and palmitic acids, precisely followed the digenic 15:1 segregation pattern, i.e., two genes control the expression of the phenotype, here it refers to mutant *ahFAD2A* and *ahFAD2B* alleles. One previous study reported that normal oleic genotype (ol_1_ol_1_ Ol_2_Ol_2_ and Ol_1_Ol_1_ Ol_2_Ol_2_) when crossed with high oleic acid genotype (ol_1_ol_1_ ol_2_ol_2_) showed monogenic (3:1) and digenic (15:1) ratio, respectively (Barkley et al., 2013). Similar to oleic acid, two other major fatty acids, namely palmitic and linoleic acids, also showed the digenic inheritance. Also, the homozygous mutant combination of both A and B subgenomes showed higher levels of oleic acid and low levels of linoleic acid and palmitic acid in the population. Although, a subgenome mutant allele had less influence on the oleic acid levels in the present study, but the combination of both mutant alleles enhanced the oleic acid levels significantly.

The correlation study showed strong negative correlation between oleic and linoleic acid. The result was further confirmed with the genotyping for the *ahFAD* mutants in the population and their respective phenotyping effect for different fatty acids. It is a well-known fact that the high oleic acid accumulates due to mutation of the *ahFAD* alleles inhibiting the conversion of oleic acid to linoleic acid (Chen et al., 2010). The variations in the content of different fatty acids by various combinations of the *ahFAD* mutant alleles were shown on the biosynthetic pathway for better understanding of the correlation between different fatty acids.

The saturated acyl chains are formed by condensation of C2 units from malonyl-acyl carrier protein to acyl chains. Each cycle adds two carbons to the growing acyl chain leading to C18:0 (stearic acid) as a major deviating point. From this C18:0, successive addition of the acyl groups leads to the formation of C20:0 (arachidic acid), C22:0 (behenic acid), and C24:0 (lignoceric acid). Furthermore, C18:0 (stearic acid) upon desaturation at C9 produces C18:1 (oleic acid), on further desaturation forms C18:2 (linoleic acid). Plants with *ahFAD* mutant alleles resulted in the accumulation of higher oleic and lower linoleic acid. As the oleic acid desaturation to linoleic was decreased, other branch for minor fatty acids synthesis like arachidic, behenic, lignoceric were increased. Interestingly, behenic acid levels were increased in plants with only homozygous *FAD* mutant alleles in both subgenomes. Stearic acid levels were not much influenced by the *FAD* mutant allele combinations and this is correlated with detection of single major QTL *qSte-I* with 78.6% PVE. Introgression of the *FAD* mutant alleles had shown an increase in the oleic acid and clearly indicated major contribution *ahFAD2B* mutant allele than the *ahFAD2A* mutant allele in the total phenotype. A homozygous combination of both mutant FAD alleles (*ahFAD2A* and *ahFAD2B*) showed accumulation of oleic acid up to 81.7%, similar to ‘SunOleic 95R,’ the high oleic parent. Genotypes with *ahFAD* allele mutation in A and B subgenomes in the homozygous state (ol_1_ol_1_ ol_2_ol_2_) showed an average increase in the oleic acid levels of 20.5%, decrease in the linoleic acid levels by 79% and palmitic acid by 20%. Although heterozygous (Ol_1_ol_1_ Ol_2_ol_2_) mutant *FAD* allele combination was influencing the major fatty acids, but in lesser extent because of a decrease in dosage of number of mutant alleles.

Although the dosage is very crucial, the major influence of mutant allele located on B subgenome on oleic, linoleic, and palmitic is very clearly seen. The presence of *ahFAD2B* mutant allele with *ahFAD2A* mutant allele showed significant variation (20% higher oleic acid, 78% lower linoleic acid, and 20% lower palmitic acid), whereas, the wild type *ahFAD2B* with mutant *ahFAD2A* allele combination showed only 0.3% increase of oleic acid, 1% decrease of linoleic acid, and 5.2% decrease of palmitic acid. This clearly indicates the importance and impact of mutant allele located in B subgenome in controlling the different fatty acids profile as compared to *ahFAD2A* mutant allele. The above results are also in agreement with the QTLs identified in these genomic regions and their effect on controlling the oleic acid phenotype. The QTLs detected at *ahFAD2B* loci showed PVE of 33.8% for oleic, 41.0% for linoleic, and 18.4% for palmitic acid, whereas QTLs identified at *ahFAD2A* mutant loci showed PVE of 17.4% for oleic, 19.5% for linoleic, and 10.7% for palmitic acid. The F_2_ plants with wild type *FAD* alleles clearly showed a decline in oleic acid and increase in the linoleic and palmitic acids. The above results indicated the importance of the dosage of the mutant *FAD* alleles on major fatty acid levels and the composition of these major fatty acids could be considered in the development of the high oleic cultivars.

### DArT and DArTseq Based Dense Genetic Map for Cultivated Groundnut

Groundnut has large and complex tetraploid genome and lack of diversity in the cultivated species decelerated the genomics studies. High marker density genetic maps are crucial for conducting high resolution QTL mapping. The first SSR based genetic map for cultivated groundnut was constructed with 135 loci (Varshney et al., 2009) and was later improved to 191 SSR loci (Ravi et al., 2011). With the application of next-generation sequencing technology, the first high density SNP (Single Nucleotide Polymorphism) based map for tetraploid groundnut with 1,685 marker loci has been constructed (Zhou et al., 2014). Recently in groundnut, a dense genetic map with the 1,152 DArT and DArTseq marker loci covering 2423.12 cM map distance was constructed for fresh seed dormancy (Vishwakarma et al., 2016). The present study reports the development of dense genetic map with 1,435 DArT/DArTseq marker loci spanning a total map distance of 1869.17 cM with an average inter marker distance of 1.3 cM. This is the most dense genetic map using DArT/DArTseq genotyping platform and the second most dense genetic map for cultivated groundnut using any of the other available marker systems.

### Genomic Regions Controlling Content of Oil and Fatty Acids

Very few studies have been conducted in the past for mapping QTLs for oil content in groundnut. With the bulk segregant analysis, PM36 SSR marker was found associated with the oil content (Selvaraj et al., 2009). Later on, based on a very less dense genetic map with 45 markers, four QTLs with PVE ranging 7.1–9.1% were reported (Sarvamangala et al., 2011). Recently for oil content in groundnut, six QTLs with 3.07–10.23% PVE were reported in the S-population and nine QTLs with 3.93–14.07% PVE were accounted in the T-population (Pandey et al., 2014). In an earlier study on six fatty acids, 164 main effect QTLs (M-QTLs) and 27 epistatic QTLs (E-QTLs) were reported in two RIL populations with the PVE ranging 0.16–40.56% (Wang et al., 2015). In our study, eight QTLs together explained 74.5% PVE and further study of these regions may provide insight into the interesting genes, which may be controlling oil content. The PVE ranging from 5.67 to 22.11% by these eight QTLs on six different LGs clearly indicated that oil content was influenced by multiple alleles spread across the genome. Similarly, 21 QTLs were detected for different fatty acids, eight of these QTLs were identified on two homologous LGs (A09 and B09) harboring the *ahFAD* mutant alleles (Pandey et al., 2014). These two LGs hold major QTLs for the three major fatty acids, namely oleic, linoleic, and palmitic acids. The clustering of the QTLs for fatty acids is common and is elucidated in groundnut (Pandey et al., 2014; Wang et al., 2015) and in several other crops, including rapeseed (Basnet et al., 2016) and soybean (Kim et al., 2010). In the present study, four clusters were reported with three QTLs each. The frequency distribution of oleic, linoleic, and palmitic acids were skewed toward one side, which showed the influence by a single factor, i.e., *FAD* alleles. The high frequency of transgressive segregations for arachidic acid and lignoceric acid might be due to complementary gene actions. The presence of transgressive segregants in lignoceric, arachidic, stearic, and behenic acids with identified QTLs on different LGs indicated the presence of specific genomic regions/genes which control the specific fatty acids. These genomic regions are very important in identifying key enzymes for targeting specific fatty acid, both to increase and decrease their content. Targeting of these clusters for further studies will be more helpful for genetic dissection, candidate gene discovery and accelerated groundnut improvement.

In summary, this study successfully developed two genetic maps based on DArT and DArTseq markers and one being the second most dense genetic map across all marker systems and most dense genetic map based on DArT and DArTseq markers in groundnut. Further, this study identified genomic regions for groundnut oil content and fatty acids. These genomic regions provide further opportunity towards gene discovery and marker development for marker-based selection to develop superior cultivars with the desired level of oil content and fatty acid profile.

## Author Contributions

Conceived and designed the experiment: RKV and MKP. Performed the experiments: YS and MKV. Analyzed data: YS, MKV, and MKP. Interpreted results: RKV, MKP, MKV, YS, and BG. Population development and phenotyping: PJ, MTV, SM, and SN. Wrote the manuscript: YS, MKV, and MKP.

## Conflict of Interest Statement

The authors declare that the research was conducted in the absence of any commercial or financial relationships that could be construed as a potential conflict of interest.

## Footnotes

1 http://www.diversityarrays.com/molecularprincip.html

2 http://www.spss.com.hk/

## Supplementary Material

**FIGURE S1 | Genetic map of F_2_ population (ICGV 07368 × ICGV 06420) segregating for oil content trait.** The mapped loci are present on the left side and their positions (in cM) are mentioned on the right side of the linkage groups.

**FIGURE S2 | Genetic map of F_2_ population (ICGV 06420 × SunOleic 95R) segregating for fatty acids.** The mapped loci are present on the left side and their positions (in cM) are mentioned on the right side of the linkage groups.

**TABLE S1 | Details of the genetic map of F_2_ population (ICGV 07368 × ICGV 06420) with information on mapped markers, linkage group, position and sequences.**

**TABLE S2 | Details of the genetic map of F_2_ population (ICGV 06420 × SunOleic 95R) with information on mapped markers, linkage group, position and sequences.**
