# Supplementary figures and images for: Molecular Mapping of Oil Content and Fatty Acids Using Dense Genetic Maps in Groundnut (Arachis hypogaea L.)
Source: Front Plant Sci. 2017 May 22;8:794. doi: 10.3389/fpls.2017.00794 (PMC5438992; doi:10.3389/fpls.2017.00794)

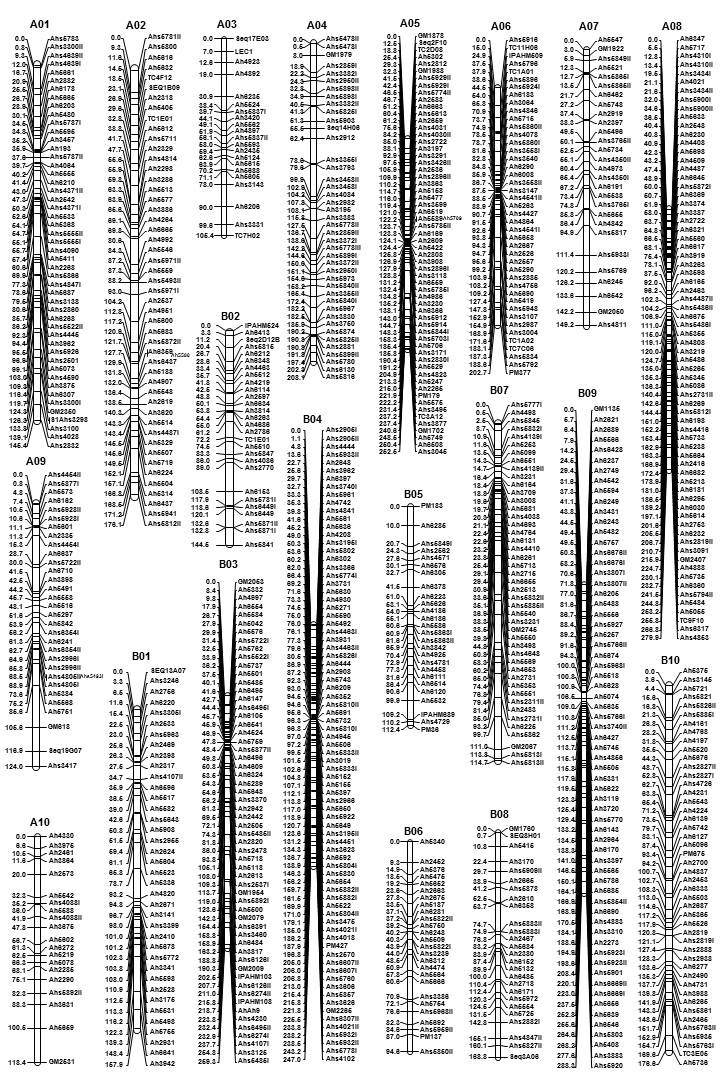

Supplement: Supplementary file 1 [file Image_1.JPEG]

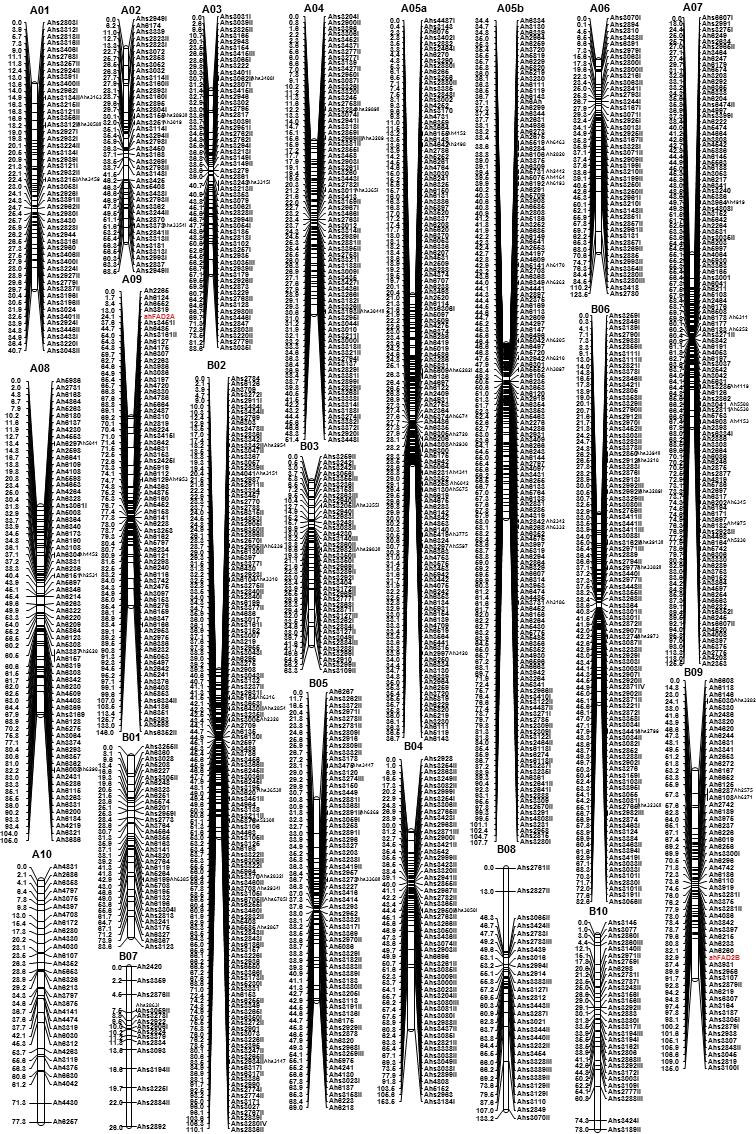

Supplement: Supplementary file 2 [file Image_2.JPEG]
